# Determinants of HIV infection among children born to HIV positive mothers on prevention of mother to child transmission program at referral hospitals in west Amhara, Ethiopia; case control study

**DOI:** 10.1186/s13052-022-01220-x

**Published:** 2022-02-03

**Authors:** Asrat Alemu, Wondwosen Molla, Kindu Yinges, Muhabaw Shumye Mihret

**Affiliations:** 1 Department of Midwifery, Dilla University, Dilla, Ethiopia; 2 School of midwifery, college of medicine and health science, University of Gondar, Gonder, Ethiopia

**Keywords:** Child HIV infection, Determinants, West Amhara, Referral hospitals

## Abstract

**Background:**

Human Immune Deficiency Virus infection among children has continued to be a global concern with an estimated 160,000 new infections in 2018. Over 90% acquire HIV from their mother. Currently, 92% of pregnant women are on antiretroviral therapy (ART). Despite, greater achievements in coverage of PMTCT and ARV drug in Ethiopia as well as in west Amhara, child HIV infections are yet an important public health problem with a high transmission rate. There are limited studies done in Ethiopia on identifying determinants of child HIV infection.

**Objective:**

This study was aimed at identifying determinants of HIV infection among children born to HIV positive mothers on the PMTCT program at referral hospitals in the west Amhara, Ethiopia, 2021.

**Methods:**

An unmatched case–control study was conducted at referral hospitals in the west Amhara region, Ethiopia. Data were collected through document review, which has been registered from July 1, 2016 to July 1, 2020. A two-stage sampling technique was applied. Consecutive sampling technique for cases and simple random sampling technique for controls was done to include a total of 320 samples (66 cases and 254 controls). Epi data 4.6 for data entry and SPSS 23 for analysis were used. Variables with p- value ≤0.2 in bivariate regression were run in the multivariable logistic regression and AOR with 95% CI and a *p*-value ≤0.05 was used to declare determinants.

**Result:**

Home delivery (AOR = 4.3; 95%CI: 2.0, 11.6), mixed feeding (AOR = 10; 95%CI: 3.2, 17.9), poor maternal ARV drug adherence (AOR = 4.3; 95% CI: 1.4, 13.4), advanced WHO clinical stage (AOR = 11.4; 95% CI: 4.1,19.1), poor nevirapine adherence (AOR = 10; 95% CI: 3.2, 22.4) and late enrollment of the infant (AOR = 15; 95% CI: 3.0, 3.0,20.5) were determinants.

**Conclusion:**

Minister of Health and NGOs should work on mobilization of the community and awareness creation on the important of exclusive breast feeding, drugs adherence, on benefit of health institutional delivery as well as the risk of homedelivery.

## Background

Mother to child transmission (MTCT) of HIV occurs when a child acquires the virus from the mother during pregnancy, delivery and / or breast feeding [1, 2]. Around 15 to 45% of the HIV positive pregnant women will transmit the virus to their children and half of these newly infected children will die before 2 years [3, 4]. MTCT accounts for more than 90% of the new child HIV infections [5]. Intervention like lifelong ART can reduce MTCT to less than 2 and 5% in non-breastfed and breastfed children respectively [6].

In 2018, around 160,000 children were newly infected with HIV and more than 100,000 died related to AIDS [7]. The vast majority of this global hit occurred in eastern and southern Africa with more than 84, 000 infections [8, 9]. In 2018, around 160,000 children were newly infected with HIV and more than 100,000 died related to AIDS [7]. The vast majority of this global hit occurred in eastern and southern Africa with more than 84, 000 infections [8, 9].

Great efforts have been made to reduce new HIV infection among children in the global as well as national level. The WHO promotes a comprehensive PMTCT approach with four prongs: primary prevention of HIV infection among reproductive age women; prevention of unintended pregnancies among women living with HIV; prevention of MTCT and providing appropriate treatment and support to mothers and children living with HIV [5]. The WHO also endorsed the option B+ approach which states that pregnant women should immediately start ART regardless of their cluster of differentiation (CD4) count and WHO clinical stage [6]. Globally, 92% of HIV positive pregnant women were on ART [10, 11], which averted around 1.4 million new child HIV infections between 2010 and 2018 [2, 12]. Comprehensive PMTCT contributed to 41% global and nearly 50% in eastern and southern African reduction in new child HIV infection from 2010 to 2018 [8, 9]. But, the new child HIV infection is yet an important public health problem and the reduction is not satisfactory to reach the 2030 global target of HIV free child [6].

As WHO priority country, Ethiopia has adopted the option B+ PMTCT approach [5] and has got significant achievement in its implementation since the start of the option B+ program in various parts of the country such as 96.5% in South Omo Zone [13], 96.1% in Hawassa [14], 94% in Addis Ababa [15] and 86.8% in Debre Birhan [16]. In 2018, an estimated 92% of the pregnant women were taking on ART and about 2700 new child HIV infection were prevented [7, 17]. Despite such a significant gain, new child HIV infection from mothers has continued to be a great challenge for the Ethiopia’s health system, with a transmission rate of 5.5% in Gondar [18], 5.8% in Bahir Dar [19], 5.9% in east and west Gojjam zones [20] and 10.1% in south Gondar zone [21]. There were limited studies done in Ethiopia on identifying determinants of child HIV infection, this study was needed because through time trend accessibility, utilization and recording of PMTCT and ARV treatment for pregnant women was increasing and changing and others used questions for study participants after identifying their HIV sero-status. This makes them prone for social desirability bias, recall bias and might raise questions on ethical acceptability of the studies since the area is ethically sensitive area. Most of other studies conducted were mainly concerned with the rate of MTCT of the HIV than identifying determinants and were lost some laboratory related important variables like CD4 count because of poor recording and laboratory by the time.

Child HIV infection due to MTCT is a global as well as a national public health problem. Child HIV infection from mother is preventable by interventions. In spite of the fact that there was a great achievement towards the coverage of PMTCT service and ARV drug utilization, child HIV infection has continued to be greater concern in Ethiopia as well as in west Amhara region with high transmission rate. Therefore, the aim of this study was to identify factors associated with HIV infection among children born to mothers on the PMTCT program of HIV and thus, the findings of this study are expected to be useful in providing effective and quality focused national PMTCT services and to inform health policy and program makers in the design and implementation of proper strategies to reduce child HIV infection from infected mother. Identified determinants of child HIV infection will be also useful to monitor the global progress towards sustainable development goal (SDG-3), 3.2 and 3.3, which aims to end preventable death of newborn and children under 5 year of age and end the epidemics of AIDS by 2030 [22].

## Methods

### Study area

The study was conducted at referral hospitals in western Amhara, Ethiopia. Amhara regional state is the second largest regional state of Ethiopia, with an area of 159,173.66 km^2^. Based on the 2007 Ethiopian central statistical agency (CSA) report, the region has a total population of over 20 million [23]. There are five referral hospitals in west Amhara region, namely Debre Markos, Felege Hiwot, Tibebe Gion, university of Gondar and Debre tabor referral hospitals. Debre Markos, Debre Tabor and University of Gondar comprehensive specialized hospitals were selected randomly by lottery method. The selected institutions were situated 130–748 km far away from Addis Ababa, the capital city of Ethiopia to the northwest. All selected hospitals are providing service for an estimated over 15 million people in the catchment areas and served as referral center for district hospitals and other health facilities. Apart from other services, the facilities included in this study are providing PMTCT service as one component of comprehensive HIV/AIDS care and support program.

### Study design and period

An unmatched case–control study design was conducted from July 1, 2016 to July 1, 2020 among children born to HIV positive mother on PMTCT program at referral hospitals in western Amhara, Ethiopia 2021.

### Study population

#### Cases

Confirmed HIV sero-status positive children at or before 24 months of age with their respective mothers who were on the PMTCT program at referral hospitals in western Amhara, Ethiopia.

#### Controls

Confirmed HIV sero-status negative children at or before 24 months of age with their respective mothers who were on the PMTCT program in referral hospitals of western Amhara, Ethiopia.

### Source population

All children at or before 24 months born to HIV positive mothers who were attending the PMTCT program at referral hospitals in west Amhara, Ethiopia.

### Study population

All children at or before 24 months born to HIV positive mothers who were attending the PMTCT program during the study period at selected referral hospitals in west Amhara region, Ethiopia.

### Sample population

All selected children at or before 24 months born to HIV positive mothers attending the PMTCT program who were enrolled to the study from the selected referral hospitals in west Amhara, Ethiopia.

### Inclusion and exclusion criteria

#### Inclusion criteria

All children with confirmed HIV sero-status at or below 24 months and whose mothers were on PMTCT program during the study period were included to the study.

#### Exclusion criteria

Children with a document which lacks important data due to transfer out or transfer in and stopped treatment follow up were excluded from the study.

### Sample size determination and sampling procedure

#### Sample size determination

In this study, sample size was calculated using Epi Info Version 7 Stat Calc function of sample size calculation for unmatched case-control study by assuming the following assumption; power of 80, 95% confidence interval (CI), 4:1 ratio of controls to cases and 6.3% of controls were exposed to abnormal breast condition and odds ratio of 5.6 [24] taken from previous similar study which gave the largest sample size of 157 children with their respective mother. Taking 5% of incomplete data set and design effect of 2, the final sample size after rounding up is 330 (66 cases to 264 controls) (Table 1).

**Table 1:** Sample size determination for factors for the study in western Amhara regional state referral hospitals, Ethiopia, 2021

Variables	Child HIV status		OR	Sample size
	Cases	Controls		
Poor ART adherence	23	40 (24%)	3.7	**140**
Abnormal breast condition	12	11 (6.3%)	5.6	**157**
Mixed breast feeding	33	10 (5.7%)	54.8	**24**

#### Sampling procedure

A two-stage sampling technique was employed. At stage one, from the five referral hospitals in west Amhara, three referral hospitals (DTRH, DMRH and UoGRH) are selected randomly by lottery method. At stage two, consecutive sampling technique was done for cases until the required sample achieved from each institution and simple random sampling technique was used to enroll controls using the child treatment card number as sampling frame and then corresponding mother’s records was obtained by using mothers’ unique ART number and/or medical record number from the selected facilities. The sample sizes was allocated to each referral hospitals proportional to the monthly client flow to their PMTCT clinic. The average monthly client flow to PMTCT clinic of each facility was 166 (33.3% of total sample size) in UoGRH, 182 (36.36% of total sample size) in DMRH and 152 (30.3% of total sample size) in DTRH. Controls were selected from the same health facility for cases.

### Variables of the study

- ❖ Dependent variable

Child HIV status (positive, negative)

- ❖ **Independent variables;** ***Child socio-demographic variables;*** Sex of the child, Age of the child at enrollment in weeks, Age of the child at confirmation of HIV status: ***Child clinical variables;*** Gestational age in weeks, Birth weight of the infant in grams, Place of delivery Duration of ROM, Feeding practice before 6 months, History of hospitalization, Feeding practice after 6 months, Immunization status of the infant, ARV prophylaxis for the infant at birth, Nevirapine adherence: ***Parental socio-demographic variables;*** Age of the mother in years, Marital status, Level of education of the mother, Religion, Place of residence, Occupation: ***Parental clinical variables;*** Mode of delivery, Duration of labor in hours, CD4 count of the mother at enrollment, WHO clinical stage of AIDS at enrollment, ANC follow-up during the current pregnancy, Time of ARV drug initiation for the mother, ART drug adherence, Maternal breast Condition, Nutritional status of the mother, Partner HIV sero-status, Partner ART enrollment

### Operational definitions

#### Confirmed HIV test result

***For breast-fed infant:*** Virology test result done for infants aged < 18 months or HIV antibody test result done 6 weeks after cessation of breast feeding for infants aged ≥18 months.

***For non-breast-fed infant****:* Virology test result done for infants aged < 18 months or HIV antibody test result done for infants aged ≥18 months [1, 25].

***ART adherence:***

ART adherence was measured according to missed doses out of 60 doses. It had three grades;

***Good:*** if missed < 3 doses out of 60 doses.

***Fair:*** if missed 3–9 doses out of 60 doses.

***Poor***: if missed > 9 doses out of 60 [1].

***Nevirapine adherence:*** was measured according to missed doses out of 60 doses. It has three grades:

***Good:*** if missed < 3 doses out of 60 doses.

***Fair:*** if missed 3–9 doses out of 60 doses.

***Poor***: if missed > 9 doses out of 60 [1, 25].

#### Nutritional status of the mother

Nutritional status was measured using Mid upper arm circumference (MUAC). It has three grades:-.

***Not malnourished***: if MUAC is greater than 22 cm,

***Moderate malnutrition:*** if MUAC is 19-22 cm and ***severe malnutrition:*** if MUAC is less than 19 cm [25].

### Immunization status of the infant

***Fully immunized:*** a child who have received one BCG, at least three doses of pentavalent vaccine, three doses of OPV and a measles vaccine.

***Partially immunized:*** a child who missed at least one dose of the 8 vaccines.

***Non-immunized:*** a child who didn’t receive any dose of the 8 vaccines [26].

### Abnormal breast condition

Maternal breast condition, which may include cracked nipples, mastitis, breast abscess, ulcer and engorgement [1].

### Mothers on PMTCT

HIV sero-status positive mothers who took the ART for prevention of child HIV infection through MTCT during pregnancy or labor and delivery or after delivery during breastfeeding [25].

### Data collection procedure

The socio-demographic characteristics and clinical profile related data of the children with their respective mother and her partner were collected from November 1, 2020 to December 1, 2020 using a data collection checklist developed from exposed infants’ care follow-up records, the integrated PMTCT registration log book and medical records of the children and their respective mother. Additional important variables were incorporated after review of literature. Data was extracted and collected by experienced Midwives on PMTCT and exposed infants’ follow up care.

### Data management and quality assurance

The data collection checklist was prepared in English and three diploma midwives and three BSc midwives were recruited as data collector and supervisor respectively. A one-day training on how to collect the data, request permission and ensure privacy and confidentiality of information was given for both the data collectors and supervisors. The supervisors supervised the data collection process and performed data quality checkups every day during the data collection. The data collection tool was pretested on 5% of the sample in Nifas Mewcha primary hospital to check for its reliability. The pretest data were not included in the main data. All collected data were checked for completeness, consistency, and clarity before entry to the software. The principal investigator oversaw the overall data collection and supervision process.

### Data processing and analysis

Data were checked, coded and entered into Epi Data version 4.6 and then exported to SPSS version 23 for cleaning and analysis. Both descriptive and analytical procedures were undertaken. Descriptive statistics like frequencies and cross-tabulations were then performed and the results were presented in texts and tables. Binary logistic regression model was computed and variables with *p*-value of ≤0.2 in the bivariate logistic regression analysis were entered in a backward likelihood ratio variable selection method to the multivariable logistic regression analysis. Multicollinearity test was done to check for significant inter-correlation between independent variables using variance inflation factor (VIF) and Hosmer and Lemeshow goodness of fit test was done to test for fitness of the model. Level of significance was declared based on the adjusted odds ratio (AOR) with 95% confidence interval (CI) at *p*-value of ≤0.05.

## Result

A total of 330 documents were reviewed, 66 cases and 264 controls. After sampling for the cases and the controls, 10 (3%) records of the controls were discarded for incompleteness and missing data. So, a total of 320 data were used for this study.

### Socio-demographic profiles of the children

Majority of study participants were males, 36 (54.5%) and 151 (57.2%) for the cases and the controls respectively. The median age of child at confirmation of HIV status was 12 months (IQR 2–18) for cases and 18 months (IQR 16–24) for controls. The median age of the infants at enrollment to PMTCT program was 8 weeks (IQR 6–18) for cases and 6 weeks (IQR 5–8) for controls.

### Clinical profiles of the children

Majority of the cases (60.6%) and the controls (89.4%) practiced exclusive breast or replacement feeding before 6 months. Fifty-eight (87.9%) of the cases and 140 (55.1%) of the controls practiced breast feeding with complementary feeds after 6 months. The Majority (60.6%) of the cases group were delivered at home. Whereas 218 (85.8%) of controls were delivered at health institution. The median birth weight of the children were 2600 g (IQR 2400–2925) for cases and 2900 g (IQR 2650–3200) for controls. The median duration of rupture of membrane for cases and the controls were 6 h (IQR 2–12) and 3 h (IQR 2–4) respectively (Table 2).

**Table 2:** Clinical profiles of children at referral hospitals in west Amhara, Ethiopia, 2021

Variables	Child HIV Status		Total (%)
	Positive (%)	Negative (%)	
**ARV prophylaxis for the infant at birth**			
Yes	25 (37.9%)	234 (92.1%)	259 (80.9%)
No	41 (62.1%)	20 (7.9%)	61 (19.1%)
**Immunization status of the child**			
Immunized	39 (59.1%)	130 (90.6%)	269 (84.1%)
Not immunized	27 (40.9%)	24 (9.4%)	51 (15.9%)
**Nevirapine adherence**			
Good	26 (39.4%)	240 (94.5%)	266 (83.1%)
Poor	40 (60.6%)	14 (5.5%)	54 (16.9%)
**Birth weight of the infant**			
Birth weight > =2500	40 (60.6%)	231 (90.9%)	271 (84.7%)
Birth weight < 2500	26 (39.4%)	23 (9.1%)	49 (15.3%)
**History of hospitalization**			
Yes	12 (18.2%)	10 (3.9%)	22 (6.9%)
No	54 (81.8%)	244 (96.1%)	298 (93.1%)

*HIV* Human immune deficiency virus*.*

### Maternal socio-demographic characteristics

The median age of mothers among the cases and the controls was 30 years (IQR 28–34.25) and 30 years (IQR 28–32) respectively. Nearly two third [26] of the cases and the majority (92.5%) of the control’s mothers were married. The majority of the cases (95.5%) and controls (94.1%) were followers of the Ethiopian Orthodox church. Majority of the controls as well as the cases mothers were urban residents, 199 (78.3%) and 36 (54.5%) respectively (Table 3).

**Table 3:** Socio-demographic characteristics of mothers at referral hospitals in west Amhara region, Ethiopia, 2021

Variables	Child HIV Status		Total (%)
	Positive (%)	Negative (%)	
**Marital status**			
Married	42 (63.6%)	235 (92.5%)	277 (86.6%)
Single	4 (6.1%)	3 (1.2%)	7 (2.2%)
Divorced	10 (15.2%)	12 (4.7%)	22 (6.8%)
Widowed	10 (15.2%)	4 (1.6%)	14 (4.4%)
**Occupation**			
Housewife	46 (69.7%)	144 (56.7%)	190 (59.4%)
Student	1 (1.5%)	6 (2.4%)	7 (2.2%)
Daily labor	10 (15.2%)	15 (5.9%)	25 (7.7%)
Government employee	4 (6.1%)	40 (15.7%)	44 (13.8%)
NGO employee	2 (3.0%)	6 (2.4%)	8 (2.5%)
Private employee	3 (4.5%)	43 (16.9%)	46 (14.4%)0
**Educational level**			
No Education	31 (47.0%)	48 (18.2%)	77 (24.1%)
Primary Education	21 (31.8%)	75 (29.5%)	96 (30.0%)
Secondary Education	7 (10.6%)	61 (24%)	68 (21.3%)
Tertiary Education	3 (4.5%)	42 (16.5%)	45 (14.1%)
Degree and above	4 (6.1%)	30 (11.8%)	34 (10.5%)

*HIV* Human Immune Deficiency Virus*, NGO* Non-Governmental Organization*.*

### Maternal clinical factors

The majority (245) of the controls and 37 (56.1%) of the cases had ANC visit. Two hundred eleven (83.1%) of controls and sixty (90.9%) of cases delivered vaginally. Fourty five (68.2%) of cases and twenty-nine (11.4%) of controls were on advanced WHO clinical stage at enrollment to PMTCT program. Almost all of controls (99.6%) and fifty-six (84.8%) of cases had normal breast condition. Almost all (98.8%) of controls and two third (44) of cases were not malnourished. The median CD4 count for the cases was 198.5 (IQR 118–323) cells/μL while that of controls was 556 (IQR 425–781) cells/μL. The median duration of labor was 14 h (IQR 8–24) for cases and 9 h (IQR 7–12) for controls (Table 4).

**Table 4:** Clinical characteristics of mothers at referral hospitals in western Amhara region, Ethiopia, 2021

Variables	Child HIV Status		Total (%)
	Positive (%)	Negative (%)	
**ARV drug adherence**			
Good	29 (43.9%)	239 (94.1%)	268 (83.8%)
Poor	37 (56.1%)	15 (5.9%)	52 (16.2%)
**Time of ART initiation**			
Before pregnancy	17 (25.8%)	197 (74.6%)	214 (64.8%)
During pregnancy	6 (9.1%)	51 (19.3%)	57 (17.3%)
During delivery	9 (13.6%)	6 (2.3%)	15 (4.5%)
During postpartum	34 (51.5%)	10 (3.8%)	44 (13.4%)
**CD4 count of mothers at enrollment (cells/μL)**			
CD4 count > 350	33 (50.0%)	218 (85.8%)	251 (78.4%)
CD4 count 200–350	8 (12.1%)	33 (13.0%)	41 (12.8%)
CD4 count < 200	25 (37.9%)	3 (1.2%)	28 (8.8%)
**Partner HIV sero-status**			
Positive	36 (54.5%)	187 (73.6%)	223 (69.7%)
Negative	16 (24.2%)	47 (18.5%)	63 (19.7%)
Unknown	14 (21.3%)	20 (7.9%)	34 (10.6%)
**Partner enrollment status (*N*** **= 223)**			
Enrolled	12 (33.3%)	163 (87.2%)	175 (78.5%)
Not enrolled	24 (66.7%)	23 (12.8%)	48 (21.5%)

### Determinants of child HIV infection

Both bivariate and multivariable logistic regression analyses was done and variables with P-value of ≤0.2 in the bivariate analysis were entered to multivariable logistic regression model with the backward Likelihood ratio variable selection method. Variables included to the final model were place of delivery, breast feeding practice, immunization status of the infant, ARV drug adherence of the mother, infant’s nevirapine adherence, mother’s CD4 count at enrollment, birth weight of infant, age of the infant at enrollment, mother’s WHO clinical stage at enrollment, ANC visit, place of residence, mode of delivery, nutritional status of the mother and breast-feeding practice after 6 months. In the final model, place of delivery (AOR = 4.3; 95% CI: 2.0, 11.6), breast feeding practice in the first 6 weeks (AOR = 10; 95% CI: 3.2, 17.9), infant’s nevirapine adherence (AOR = 10; 95% CI: 3.2, 22.4), ARV drug adherence of the mother (AOR = 4.3; 95% CI: 1.4, 13.4), mother’s WHO clinical stage enrollment (AOR = 11.4; 95% CI: 4.1,19.1) and Age of the infant at enrollment (AOR = 15; 95% CI: 3.0, 20.5) were declared to have statistically significant association with child HIV infection (Table 5).

**Table 5:** Determinants of HIV infection among children born to HIV positive mothers on PMTCT at referral hospitals in west Amhara, Ethiopia, 2021

Variables	Child HIV Status		COR (95% CI)	AOR (95% CI)
	Positive	Negative		
**Place of delivery**				
Home	40	36	9.3 (5.0, 14.0)	4.3 (2.0, 9.6) **
Health Institution	26	218	**1**	
**Place of residence**				
Rural	30	55	3.0 (1.7, 5.3)	
Urban	36	199	**1**	
**ANC visit**				
No	22	16	7.437 (2.4, 8.40)	
Yes	44	238	**1**	
**Mode of delivery**				
Vaginal Delivery	60	211	2.0 (1.8, 5.0)	
Cesarean Delivery	6	43	**1**	
**Breast feeding practice in the first six weeks**				
Mixed feeding	26	27	5.5 (2.9, 10.0)	10 (3.2, 17.9) ***
EBF/ERF	40	227	**1**	
**Immunization status of the infant**				
Non immunized	27	24	6.6 (3.5, 12.6)	
Immunized	39	230	**1**	
**ARV prophylaxis for the infant at birth**				
Not given	41	20	19.2 (6.8, 27.7)	
Given	25	234	**1**	
**Infant’s nevirapine adherence**				
Poor	40	14	26.4 (9.7, 34.8)	10 (3.2, 22.4) ***
Good	26	240	**1**	
**Maternal ARV adherence**				
Poor	37	15	20.3 (9.9, 23.1)	4.3 (1.4,13.4) **
Good	29	239	**1**	
**Nutritional status of the mother**				
Malnourished	22	3	41.8 (12, 145)	
Not malnourished	44	251	**1**	
**Mother’s WHO clinical stage at enrollment**				
WHO Stage > 1	45	29	16.6 (8.7, 21.7)	11.4 (4.1,19.1) ***
WHO Stage 1	21	225	**1**	
**Mother’s CD4 count at enrollment**				
CD4 </= 350	33	36	6.0 (3.3, 11.0)	
CD4 > 350	33	218	**1**	
**Age of the infant at enrollment**				
Enrolled < 6 weeks	32	12	18.9 (6.9, 29.4)	15.0 (3.0, 20.5) **
Enrolled = < 6 weeks	34	242	**1**	
**Birth weight at delivery in grams**				
Birth weight < 2500	26	23	6.5 (3.4, 12.5)	
Birthweight > = 2500	40	231	**1**	

** *p*-value < 0.01, *** *p*-value < 0.001, *EBF* exclusive breast feeding, *AOR* adjusted odds ratio, *COR* Crude odds ratio,

## Discussion

Mother to child transmission of HIV has continued to be the main route of HIV transmission to children, in turn AIDS is an important contributor to childhood morbidity and mortality in Ethiopia. So, identifying the significant factors to child HIV infection can be important to reduce AIDS related morbidity and mortality. This study assessed child and maternal socio-demographic and clinical characteristics and their association with child HIV infection from mother. Accordingly, home delivery, mixed breast-feeding practice, poor infant nevirapine adherence, poor maternal ART adherence, mother’s advanced WHO clinical stage at enrollment and late enrollment of child became determinants for child HIV infection.

In this study, the odds of being HIV positive among home delivered children was 4 times more likely compered to children who are born at health institution. This finding is consistent with the study conducted in Uganda [27] west Shewa zone [28] and Dire Dawa Ethiopia [29]. The possible reason for this might be due to that a women who gave birth at home will not get PMTCT service like active management of labor with pantograph, infection prevention precautions, safe delivery practice and infant ARV prophylaxis at birth which are available at the health institution [1].

HIV-exposed children who had been mixed breastfed were 10 times more likely to get infected with HIV than those who had been exclusive breastfed or replacement fed. This finding is consistent with the studies done in Zimbabwe [30] Kenya [31] Abidjan, Cote d’Ivoire [32], Assela [33], Addis Ababa [24], southwest Ethiopia [34], Dire Dawa [29] and southern Ethiopia [35]. The possible reason for this might be breast milk is the ideal food for newborns and infants in the first 6 months due to their immature gastrointestinal system. So that feeding the infant with additional foods than only breast milk can cause irritation and ulceration of its gastrointestinal tract, which may lead to easy communication between maternal breast milk and infant’s blood stream to end up in transmission of the virus [1].

Compared to HIV-exposed children from a mother who had good adherence to ART, those HIV-exposed children from a mother who had poor adherence were more than 4 times more likely to get infected with HIV. This finding is consistent with the study conducted in rural Kenya [31] and Addis Ababa Ethiopia [24]. The possible explanation might be that ARV drugs play crucial role in reducing maternal viral load. So that viral load would be very high in those mothers with poor ARV drug adherence, which in turn increases the chance of MTCH [25].

Compared to HIV-exposed infants who had good nevirapine adherence, those HIV-exposed infants who had poor nevirapine adherence were 10 times more likely to get infected with HIV. This finding is consistent with similar the study done in Kenya [31] and supported by the study from Zimbabwe [36]. It is scientifically accepted fact that good adherence to nevirapine for infants decreases the risk of HIV infection among exposed children [2].

Those HIV exposed infants enrolled lately were 15 times more likely to get infected than those HIV exposed infants enrolled early. This finding is consistent with study which was done in Cameroon [37], Nigeria [38] and northwest Ethiopia [39]. The possible explanation might be those infants enrolled lately were more likely to be those who were sick, had depressed immunity by childhood illnesses and couldn’t been on ARV drug prophylaxis.

Compared to HIV exposed children from mothers with WHO clinical stage one, those HIV exposed children from mothers with advanced WHO clinical stage were more than 11 times more likely to get infected. This finding is in line with finding from Addis Ababa [24] and Oromia regional state [40]. The possible explanation might be advanced WHO clinical stage of HIV indicates more opportunistic infections and malnutrition leading to further immunodeficiency and associated high viral load.

### Limitations of the study

The finding of this study should be interpreted in light of some limitations. The retrospective nature of data collection might have data incompleteness in some of children’s and mother’s document and might have some measurement error.

## Conclusion

Home delivery, mixed feeding practice, poor maternal ARV drug, poor nevirapine adherence, late enrollment of Infant, and advanced WHO clinical stage at enrollment were all determinants of HIV infection among children born to HIV positive mothers in this study.

Ministers of Health, Regional Health Offices, Zonal Health Departments, and NGOs working on HIV programs should work on community mobilization and awareness creation about the importance of exclusive breast feeding, ARV drug adherence for mothers and nevirapine adherence for infants, the benefits of health institutional delivery as well as the risks of home delivery by giving special attention to mothers with advanced WHO clinical stage and infant enrolled lately as high risk in order to reduce vertical transmission of HIV and contribute to the goal of an AIDS-free generation by 2030. Furthermore, referral hospitals should establish a large network with nearby health institutions in order to reduce home deliveries and effectively counsel on exclusive breast feeding during ANC and PMTCT follow-up. Finally, in order to reduce the risk of vertical transmission, health policymakers and program developers should develop and implement a special strategy based on the identified determinants.

## Data Availability

All data included in this manuscript can be accessed from the corresponding author upon request through the email address.

## Abbreviations

AIDS: Acquired immune deficiency syndrome
ANC: Antenatal Care
ART: Antiretroviral therapy
ARV: Antiretroviral
CD4: Cluster of differentiation
CI: Confidence interval
DNA: Deoxyribonucleic acid
FMoH: Federal Ministry of Health
HEI: HIV exposed infant
HIV: Human immune deficiency virus
MTCT: Mother To Child Transmission
NVP: Nevirapine
OR: Odds Ratio
SPSS: Statistical Package for Social Sciences
STI: Sexually Transmitted Infection
USAIDS: The Joint United Nations Program on HIV/AIDS
WHO: World Health Organization
