# Correction to: Determinants of HIV infection among children born to HIV positive mothers on prevention of mother to child transmission program at referral hospitals in West Amhara, Ethiopia; case control study

**DOI:** 10.1186/s13052-022-01230-9

**Published:** 2022-02-24

**Authors:** Asrat Alemu, Wondwosen Molla, Kindu Yinges, Muhabaw Shumye Mihret

**Affiliations:** 1 Department of Midwifery, Dilla University, Dilla, Ethiopia; 2 School of Midwifery, College of Medicine and Health Science, University of Gondar, Gonder, Ethiopia

**Correction to: Italian J Pediatr 48, 1–9 (2022)**

**https://doi.org/10.1186/s13052-022-01220-x**

The original article omitted part of co-author, Muhabaw Shumye Mihret’s name which has since been re-instated.

